# ART access-related barriers faced by HIV-positive persons linked to care in southern Ghana: a mixed method study

**DOI:** 10.1186/s12879-016-2075-0

**Published:** 2016-12-07

**Authors:** Augustine Ankomah, John Kuumuori Ganle, Margaret Yaa Lartey, Awewura Kwara, Priscilla Awo Nortey, Michael Perry Kweku Okyerefo, Amos Kankponang Laar

**Affiliations:** 1 Department of Population, Family and Reproductive Health, School of Public Health, University of Ghana, Legon, Accra, Ghana; 2 Department of Medicine, University of Ghana School of Medicine & Dentistry, University of Ghana, Legon, Accra, Ghana; 3 Department of Medicine, Warren Alpert Medical School of Brown University, Providence, RI USA; 4 Department of Epidemiology and Disease Control, School of Public Health, University of Ghana, Legon, Accra, Ghana; 5 Department of Sociology, University of Ghana, Legon, Accra, Ghana

**Keywords:** HIV, AIDS, PLH, Antiretrovirals, Antiretroviral therapy, Access, Barriers, Ghana

## Abstract

**Background:**

Timely and enduring access to antiretroviral therapy (ART) by HIV-infected individuals has been shown to substantially reduce HIV transmission risk, HIV-related morbidity and mortality. However, there is evidence that in addition to limited supply of antiretrovirals (ARVs) and linkage to ART in many low-income countries, HIV+ persons often encounter barriers in accessing ART-related services even in contexts where these services are freely available. In Ghana, limited research evidence exists regarding the barriers HIV+ persons already linked to ART face. This paper explores ART access–related barriers that HIV+ persons linked to care in southern Ghana face.

**Methods:**

A mixed method study design, involving a cross-sectional survey and qualitative in-depth interviews, was conducted to collect data from four healthcare providers and a total of 540 adult HIV+ persons receiving ART at four treatment centres in Ghana. We used univariate analysis to generate descriptive tabulations for key variables from the survey. Data from qualitative in-depth interviews were thematically analysed. Results from the survey and in-depth interviews were brought together to illuminate the challenges of the HIV+ persons.

**Results:**

All (100%) the HIV+ persons interviewed were ARV-exposed and linked to ART. Reasons for taking ARVs ranged from beliefs that they will suppress the HIV virus, desire to maintain good health and prolong life, and desire to prevent infection in unborn children, desire both to avoid death and to become good therapeutic citizens (abide by doctors’ advice). Despite this, more than half of the study participants (63.3%) reported seven major factors as barriers hindering access to ART. These were high financial costs associated with accessing and receiving ART (26%), delays associated with receiving care from treatment centres (24%), shortage of drugs and other commodities (23%), stigma (8.8%), fear of side effects of taking ARVs (7.9%), job insecurity arising from regular leave of absence to receive ART (5.3%), and long distance to treatment centres (4.9%).

**Conclusions:**

The results in this study suggest that efforts to provide and scale-up ART to all HIV+ persons must be accompanied by interventions that address structural and individual level access barriers.

**Electronic supplementary material:**

The online version of this article (doi:10.1186/s12879-016-2075-0) contains supplementary material, which is available to authorized users.

## Background

Globally, timely access to antiretrovirals (ARVs) and antiretroviral therapy (ART) by people living with HIV (PLH) has been shown to substantially reduce morbidity and mortality, reduce HIV transmission risk even with modest ART coverage of the HIV-infected population and imperfect ART adherence, improve employment rates to levels prior to HIV infection, as well as improve the overall quality of life of PLH [1–5]. Indeed, one recent report detailed the benefits of ART as follows: ART averted 5.5 million deaths in low- and middle-income countries between 1995 and 2012, with Sub-Saharan Africa accounting for most of those lives saved; ART reduced the risk of HIV transmission by up to 96% and the risk of tuberculosis infection among PLH by 65% [6]. Also, spending on ART generated economic returns of double or more than the initial investment, while working-age adults living with HIV can return to work earlier when they receive treatment, boosting labour productivity and reducing hardship among affected households [6].

These benefits notwithstanding, durable access to ART remains a crucial problem for PLH in resources-limited settings, and studies continue to highlight the challenges in ensuring effective HIV continuum of care (i.e. Proportion who know their diagnosis of HIV, of them how many engaged in HIV care, of them how many prescribed ART, retained on ART, and of them proportion with viral load undetectable) [4–7]. There is evidence that in addition to limited supply of ARVs and ART in many low-income countries, PLH often encounter challenges in accessing ARVs and ART services even in contexts where these services are freely available [4–7]. For example, although the number of people receiving ART continues to rise in Africa, it is estimated that approximately three-quarters of adults living with HIV in sub-Saharan Africa have no access to ART [6, 8]. Of the 21.2 million people in Africa eligible for ART in 2013 based on the 2013 WHO guidelines for example, only 7.6 million people were receiving HIV treatment [6]. In the case of Ghana, ART for PLH was piloted in June 2003 [9]. The Ghana National HIV/ STI Control Programme of the Ghana Health Service reported that by December 2007, only about 13,429 out of an estimated 70,000 HIV+ persons who needed ART had been put on treatment [10]. As at November 2009 the number of HIV+ persons who were receiving ART rose to about 31,400 [11]. Despite this laudable achievement in Ghana, about 70% of PLHIV in need of ART did not have access in 2009 [11].

Gaps in testing, delays in seeking/accessing treatment and retention are acknowledged weaknesses the HIV prevention-care continuum in Ghana. Successful attainment of treatment cascade steps has not been fully achieved. For example, HIV counseling & testing in the general adult population remains low, within the range of 4–10% [11]. Of note, while still lower than the optimal level, counseling and testing among pregnant women is up to 67% as of 2011. Despite these gains, about half of eligible people living with HIV received ART in 2011 [11]. However, data on adherence to ART regimens is poor likely due to the difficulties in linking HIV+ persons to treatment as well as retention into care. Reliable estimates on achievement of the key HIV care cascade steps have not been widely reported in Ghana. The corresponding author of this paper and his team are currently implementing a study to understand the barriers as well as facilitators to achieving the HIV treatment and prevention care cascade in Ghana.

In the international research literature, a number of barriers have been identified as hindering access to ART. Limited trained health staff [12–14], lack of comfortable hospital facilities to provide ART [15, 16], shortages and unavailability of ART in certain places especially in low-income settings [17, 18], high costs, including travel, opportunity and social costs associated with ART [7, 19], are some of the most commonly cited barriers HIV+ persons face in accessing ART. Fear of side effects [20], fear of stigma and discrimination [14, 21] and lack of adequate social support [22] have also been reported as formidable barriers hindering access to ART.

While there is growing recognition in both international and local policy and empirical research literature that PLH may encounter barriers in accessing ART services even in contexts where these services are freely available, there is limited research in Ghana that asks PLH about the barriers that PLH already linked to ART face. For example, one study in Ghana suggested that shortage of ARVs and differential pricing policy between private and public health facilities - the ART services in private health facilities attract a fee of about $30 per month, ten times greater than the public rate - hindered access to ART [11]. Another study suggested that long waiting time before care is received in ART clinics discouraged PLH from accessing ART in Ghana [9]. While these pioneering studies in Ghana have highlighted some of the barriers to accessing ART, they nevertheless have not paid sufficient attention to the perspectives of PLH. Understanding the perspectives of PLH in relation to the barriers they face in accessing ART could however form the basis for implementing contextually relevant programmatic initiatives to both redress these challenges and better improve access and adherence to ART [14]. This paper presents and discusses access –related barriers that HIV+ persons already linked to ART in southern Ghana face.

## Methods

### Study design

This study forms part of a larger original study that was conducted to examine non-prescription drug use among HIV+ persons on ART, adherence to ART, and barriers HIV+ persons face accessing ART in Ghana. A mixed method study design, involving a cross-sectional survey and qualitative in-depth interviews, was conducted to collect data from healthcare providers and a total of 540 adult HIV+ persons receiving ART at four treatment centres in the Eastern and Greater Accra regions of Ghana. For the purposes of the current paper, we focus on, and report findings from both the survey and qualitative interviews that are related to the barriers HIV+ persons face accessing ART.

#### Study sites

Empirical research was conducted at four health facilities in southern Ghana where ART is offered to HIV-infected clients. These health facilities are the Fevers Unit of the Korle Bu Teaching Hospital and the Tema General Hospital (both in the Greater Accra region of Ghana), and the Atua Government Hospital, and St Martins de Porres Hospital in the Eastern region of Ghana. The Korle Bu Teaching Hospital is one of the tertiary hospitals in southern part of Ghana. The Fevers unit is affiliated to the department of medicine. This unit treats cases such as measles, rabies, chicken pox, tetanus and chronic diarrhoea among others. At the time of the study, the Fevers Unit of the Korle Bu Teaching Hospital had about 6000 patients who were on ART. Tema General Hospital sees about 1500 HIV+ clients who are enrolled on ART. The Atua Government Hospital and St. Martins de Porres Hospital were the first PMTCT sites in Ghana. The Atua Government hospital and the St. Martins de Porres hospital have about 4800 and 4000 HIV+ clients on ART respectively.

### Study participants

The study participants comprised adult HIV+ persons receiving ART at the Korle Bu Teaching Hospital, the Tema General Hospital, the Atua Government Hospital and the St. Martins de Porres Hospital. In addition, healthcare providers (i.e. Nurse Prescribers who manage the ART clinics) at the four study sites were interviewed.

### Sample size estimation and sampling

The sample size for the survey component of the study was determined using Statcalc in Epi Info 2000 package. The exact number of HIV+ persons on ART who may be experiencing our primary outcome of interest (i.e. face barriers accessing ART) is unknown in the four study sites. Based on previous literature [9, 14] we estimated that 50% of the total number of HIV+ persons on ART from the four study sites face one barrier or the other accessing ART. With an alpha of 0.05 and a statistical power of 90%, 434 clients were computed as the minimum sample needed to assess the barriers HIV+ persons face accessing ART from the four treatment centres. To account for non-response and errors in data recording, this sample size was further increased by 20%. The final sample size was thus 434 + 87 = 521. However, being part of a larger study with an overall sample size of 540, all of the 540 PLH were used for this analysis.

Prior to sampling, the probability proportional to size weighting procedure was employed in the allotment of the HIV+ persons to the four study sites. Study participants were selected using systematic random sampling procedures. To do this, the master list of ART clients at each facility served as the site-specific sampling frame. The sampling interval (n) for each site was derived by dividing the total number of participants on the register by the required sample at each site.

For the qualitative component of the study, we purposively selected four ART In-charges (one each from the four study sites) for in-depth interviewing. These ART In-charges are healthcare providers (usually Nurse Prescribers) who manage the ART clinics at their respective hospitals. Therefore, not only were they knowledgeable in the workings of ART, but also they knew most of the HIV+ persons who were on ART in their respective clinics.

### Study instruments

For the survey component of the study, questionnaires were the data collection instruments. The questionnaire had closed-ended questions as well as open-ended questions. The questionnaires were designed to elicit information on the various aspects of ART, including the socio-demographic characteristics of ART clients, desire and motivation to be on ART and barriers encountered in accessing ART. Apriori identified potential barriers to accessing ART, which formed part of the questionnaire, included periodic shortages of ARVs, cost of ARVs, side effects associated with ARVs, and perceived efficacy of alternative medications.

For the in-depth interviews with the four ART In-charges, an open-ended thematic topic guide was designed and used as the data collection instrument. The instrument was designed to ensure that similar themes and questions were covered in each interview. The instrument however had built-in flexibility that allowed for any pertinent but unexpected issues that arose during the individual interview process to be further probed. The instruments focused on exploring nurse priscribers’ perspectives on HIV+ persons’ motivations for staying on ART, the nature of barriers encountered in accessing ART and how these barriers affected access to ART.

To ensure reliability, the study instruments were pre-tested on HIV+ persons on ART who were not selected for the actual study. The needed corrections and validations were done prior to the actual data collection.

### Data collection

The total number of respondents needed from a particular study site was interviewed between May 5 – June 30 2014. With the help of the ART Programme In-Charges, eligible study participants were identified and interviewed using paper-based questionnaires and topic guides. Interviews were conducted by 8 research assistants (RAs), and supervised by the PI. Typically, each questionnaire interview session took approximately 40 min, and participant’s responses to questions were recorded on questionnaires. In-depth interviews however lasted 40 - 60 min, and the discussions were audio tape-recorded. In addition to conducting interviews, the RAs also reviewed participants’ hospital records and relevant data were extracted.

The RAs were recruited from the University of Ghana’s School of Public Health based on experience of conducting surveys and in-depth interviews as well as knowledge of the local area. They were all trained. Training entailed introduction to the study objectives, goals, methods and expected outcomes. There were extensive role-plays to ensure accuracy during data collection.

### Data management and analysis

Data were reviewed daily for inconsistencies or omissions and problems with data completion addressed. Data collectors reviewed all completed data collection forms and corrected any errors or inconsistencies before hand-delivering them, along with their accompanying consent forms to field supervisors. Supervisors also reviewed the forms for accuracy, consistency, and completion. Once the data collection forms were considered complete, they were securely delivered to the principal investigator’s office where they were kept in locked filing cabinets. All quantitative survey data were doubly entered into pre-programmed data screens designed in CSPro (Census and Survey Processing System) and doubly entered at a centralised location at the PI’s institution. Data were exported to IBM SPSS Statistics, version 20, for consistency checks and validation. Cleaned and validated data sets were analysed using IBM SPSS Statistics. Analyses of survey data were descriptive; we used univariate analysis to generate descriptive tabulations for key variables from the survey.

For the qualitative data, audio tape recordings of in-depth interviews were transcribed and the data were thematically analysed. Results from the survey and in-depth interviews were then brought together to illuminate the barriers HIV+ persons face accessing ART in Ghana.

## Results

### Characteristic of study participants

A total of 540 questionnaires were returned after the survey. Our unit of analysis is therefore 540. Table 1 shows the characteristics of the 540 ART clients who completed the survey. The mean age of the study participants was 42.3; age ranged from 18 to 65 years. Majority (74.1%) of the participants were females.

**Table 1:** Characteristic of study participants (*n* = 540 unless indicated otherwise)

Characteristic	Frequency	Percent
Study site		
Atua Government Hospital	146	27.0
St Martin’s Martins de Porres Hospital	148	27.4
Tema General Hospital	93	17.2
Fevers Unit, Korle Bu Teaching Hospital	153	28.3
Region		
Greater Accra Region	246	45.6
Eastern Region	294	54.4
Place of residence		
Urban	275	50.9
Rural	265	49.1
Sex of respondent		
Male	140	25.9
Female	400	74.1
Marital status of respondent		
Single	82	15.2
Married	231	42.9
Divorced/Separated	78	14.5
Widowed	108	20.0
Cohabiting	40	7.4
Religious affiliation of respondent		
Not religious	10	1.9
Christian	485	89.8
Muslim	43	7.9
Traditionalist	2	.4
Level of education		
No formal education	109	20.1
Primary	123	22.9
JHS	170	31.4
SHS/Vocational	102	18.9
Postsecondary /Tertiary	36	6.7
Current Occupation		
Unemployed	107	21.0
Self-employed Farmer	338	66.4
Formal sector employment	9	1.8
Other	55	10.8
Total	509	100.0
Age^a^		
18–19	5	.9
20–24	12	2.2
25–35	126	23.3
36–60	374	69.3
61+	23	4.3
HIV Disease State		
HIV positive with no AIDS	365	67.6
HIV positive with AIDS	175	32.4
Number of years on ART		
Don’t know	40	7.4
< 1	47	8.7
1–3	171	31.7
4–6	152	28.1
7–10	117	21.7
11 and above	13	2.5
CD4 Cell Count, median (range)	373 (2-1663.0)	
Total	540	100

^a^Mean age is 42.3; age ranged from 18 to 65 years

The majority (42.9%) of the participants were also married. Christians constituted 89.8% of the total sample, while 20.1% of the participants had no formal education. Also, majority (66.4%) of the participants were self-employed farmers. Except 7.4% of the participants who did not know how long they have been on ART, the length of time most participants reported being on ART ranged from 1 month to more than 11 years.

### Desire and motivation to be on ART

Before examining the barriers PLH face accessing ART, we first investigated participants’ desire and motivation to be on ART. Results from the survey suggest that all (100%) the HIV+ persons interviewed were already linked to ART. Findings from qualitative open-ended questions embedded in the survey reveal a number of reasons why all participants desire to be on ART. These range from beliefs that ART will suppress the HIV virus, desire to maintain good health and prolong life, and desire not to infect unborn children, to desire to avoid death as well as desire to become a good therapeutic citizen (abide by doctors’ advice). One female participant for example said: *‘I want to be on ART because the ART will suppress the HIV virus. This will help me maintain good health and prolong my life’*. One male participant also said: *‘I want to be on ART…I take the drugs because I do not want to die early. I take the drug to prolong my life so as to be able to look after my children’*. One other female participant related why she was on ART and why she wants to be on it: *‘Without ART, I would have been dead by now. It keeps me alive. That is why I want to be on it’*. Another female participant said: *‘We are asked at the counseling session to take our drugs…to take only the doctor’s medicine. I do not want to disobey the doctor’*. One male participant also said: *‘I am on ART because I do not want to infect my unborn child’*.

Indeed, interviews with ART Programme In-charges corroborated the accounts HIV+ persons gave. The ART Programme In-Charge at the ART clinic at Korle Bu hospital makes the point like this:

*‘I think they [referring to ART clients] want to be on ART because they do not want to joke with their lives. They value their lives and some of them too when they started coming to the ART clinic, the way they felt, the way they were sick and how they have improved have made them believe that indeed the ARVs work’*.

The ART Programme In-Charge of the ART clinic at Atua government hospital shared similar views:

*‘I think many of our clients are on ART and do want to be on ART because they have realised the dangers of not being on ART. Most of them have realised that they can lead normal lives and be able to have children who are HIV-free. So they are very motivated to come for treatment’.*

Most of the narrative accounts that both the HIV+ persons and ART Programme In-charges gave suggested that most ART clients in the four study sites not only recognise the dangers of not being on ART, but they also recognise the benefits of being on ART. It is therefore the recognition of dangers and benefits that co-mingled to motivate most participants to stay on ART.

### Barriers to accessing ART

Despite the fact that all the participants in this study expressed a desire to receive and remain on ART, a number of barriers were reported as hindering access to ART. Table 2 shows a tabulation of responses to three questions that asked participants to indicate three barriers (in order of priority) they face accessing ART. Seven main first priority barriers were reported by 63.3% of all the study participants. These were delays associated with receiving care from treatment centres (10.9%), long distance to treatment centres (1.1%), high financial costs associated with accessing and receiving ART (12.6%), job insecurity arising from regular leave of absence to receive ART (2%), shortage of drugs and other commodities (13.3%), fear of side effects of taking ARVs (4.8%), and stigma (1.3%).

**Table 2:** Barriers encountered in accessing ART

Barrier	1^st^ Barrier		2^nd^ Barrier		3^rd^ Barrier		Total
	Freq	%	Freq	%	Freq	%	*N*
Delay before care is received	59	10.9	35	6.5	9	1.7	103
Long distance to treatment centres	6	1.1	24	4.5	11	2	41
Financial challenges	68	12.6	24	4.4	19	3.5	111
Job insecurity	14	2.6	7	1.3	5	0.9	26
No challenge	198	36.7	350	64.8	418	77.4	966
Shortage of drugs and other commodities	72	13.3	34	6.3	12	2.2	118
Fear of side effects	26	4.8	8	1.5	2	0.4	36
Stigmatisation	7	1.3	6	1.1	3	0.6	16
Total	540	100	540	100	540	100	1620

The percentage of participants reporting delays associated with receiving care from treatment centres, long distance to treatment centres, high financial costs associated with accessing and receiving ART, job insecurity arising from regular leave of absence to receive ART, shortage of drugs and other commodities, fear of side effects of taking ARVs, and stigma as second priority barriers however decreased to 6.5, 4.5, 4.4, 1.3, 6.3, 1.5, and 1.1% respectively. These percentages, respectively, further decreased to 1.7, 2, 3.5, 0.9, 2.2, 0.4, and 0.6% when participants were asked to indicate their third priority barrier. Crucially, 36.7% of the participants reported that they did not face any barriers accessing ART when they were asked to mention their first priority barrier. This percentage, respectively, increased to 64.8 and 77.4% when participants were asked to indicate their second and third priority barriers.

Responses from the qualitative open-ended questions embedded in the survey tools and the four ART Programme In-charges illuminated the nature of some of the barriers and the ways in which these barriers acted to hinder access to ART for different categories of HIV+ persons. In relation to the cost associated with ART for example, many of the participants reported that although government has subsidised the costs of ARVs, the five Ghana cedis that they pay monthly to receive ARVs was still not affordable, especially for those who are not employed. Coupled with costs associated with transportation as well as opportunity costs of travelling to receive care, several participants, especially from rural areas, reported that they are sometimes deterred from seeking ART because of financial constraints. One female participant makes the point thus:

*‘I have challenges with paying for the drugs. We pay monthly five cedis for the drugs; though this is not much, I am unemployed and I do not have this amount all the time. Also, because I come from the village, I need money to pay for transportation. They will refuse to give me the drugs too if I do not have the money to pay. This is my number one challenge’.*

Another female participant also related how delay associated with receiving care from ART clinics was an important barrier to ART access*. ‘My major problem is the long waiting time…sometimes we go early to the clinic but you will come home very late. This makes me feel reluctant to go sometimes’.* Many of the participants felt that they spend too much time waiting for the needed services at each clinic visit. Delays at ART clinic were reported to come from three main sources, namely delay before folder is received, delay before seeing a doctor/counselor, and delay at the pharmacy where medicines are dispensed. These delays were particularly concerning for participants who are employed in the formal sector, and who often need permission to absent themselves from work in order to attend ART clinic sessions. As one participant said: *‘My boss does not want me out of the factory for long during working hours’*. Thus long hours of absence from work was reported to breed job insecurities as employers are more likely to replace workers who frequently demand leave of absence. Indeed, a number of participants made reference to HIV+ persons who had lost their jobs due partly to their HIV+ status, arising from regular requests for leave of absence to seek treatment.

Others also spoke about how regular shortages of drugs and other commodities such as nutritional supplements often serve as disincentive for visiting ART clinics. One male participant made the point like this:

*The problem I have encountered is that sometimes I will travel from my village to the clinic and they will say there is no medicine or they will give me small medicine and ask me to come the next week. You know it is not easy to travel all this long distance.*

Some participants also reported how fear of side effects and stigmatisation were major barriers to accessing ART. ‘*I experience some rashes on my skin which do not allow me to come for medicine’.* Participants who were on ART for a relatively shorter time period before this study, particularly reported side effects such as rashes. Several of the narratives here suggested that the physical manifestation of these side effects often triggered questions and suspicions from both family members and the general public. In relation to stigma, one participant said: *‘My heart beats faster whenever it is time for me to go for ART…I am always afraid that I will meet someone I know at the clinic…you know the stigma’.* Thus fear of being seen in an ART clinic by a known family member or friend and the fear that one maybe stigmatised acted as serious disincentives for HIV+ persons to access and use ART services.

Results from in-depth interviews with the ART Programme In-charges largely supported a number of the barriers HIV+ persons reported. For instance, the ART Programme In-Charge at the ART clinic at the St. Martins de Porres hospital noted that:

*‘Sometimes they* [referring ART clients] *are poor and they don’t even have food to eat. How then can such people pay for the transport and the small fees that they are required to pay every month before receiving treatment?’*

The ART Programme In-Charge at the ART clinic at Korle Bu hospital did not however think cost should be a hindrance to accessing ART:

*‘HIV treatment is basically free here. They* [referring to ART clients] *don’t pay for consultation. The medicines, they pay a token of GHS 5 a month. The medicine itself is free but the GHS 5 a month is to help the administrative work and even with that, now they’ve introduced health insurance into it, so those who have health insurance, it covers that GHS 5 so they end up not paying and if you come and you don’t have health insurance and GHS 5, you are still given your medicine but the medicine you don’t pay for it. So I don’t think cost should be a problem’.*

This participant however acknowledged that periodic shortage of drugs and basic consumables was a challenge.

*‘At times there is shortage of drugs and when there is shortage of drugs, it becomes a headache for us because we don’t know what is going to happen to the patients. We ourselves when there is shortage of drugs, test kits and other things, it is very difficult for us to work’.*

## Discussion

This paper adds to the small but growing number of empirical studies in the Ghanaian context that have attempted to explore the barriers HIV+ persons face in accessing ART. The paper reveals a number of findings that should not be overlooked. The majority of our study participants were either self-employed farmers (66.4%) or unemployed (21%). Previous literature has highlighted the causally-causative nature of the relationship between poverty, unemployment and HIV/AIDS infection: that poverty and unemployment could expose individuals to risks of HIV/AIDS infection just as being HIV/AIDS positive could trigger poverty and unemployment [23]. While our study did not set out to investigate this relationship, in-depth interviews with some participants in this study suggested that in some cases, self-employment and unemployment were themselves the product of HIV+ persons losing their jobs in other viable sectors of the economy due partly to their HIV+ status. Self-employment in farming is an important livelihood activity in Ghana; but evidence from our qualitative in-depth interviews suggested that most of the study participants were subsistent farmers or were poor. This could potentially limit the ability of such persons to generate and accumulate sufficient stock of resources to be able to access ART as well as meet their nutritional requirements. This would suggest the need for targeted social and economic safety net interventions such as conditional cash transfer programmes and free or subsidised nutritional supplementation interventions to support unemployed and poor HIV+ persons to pay for HIV treatment costs and meet their daily nutritional requirements. In the context of Ghana more specifically, it might be beneficial to include unemployed and poor PLH in current social protection programmes such as the Livelihoods Empowerment Against Poverty (LEAP) programme.

Findings also revealed that 74% of the study’s participants were women. This statistic, we believe, is not the result of sampling bias as both our examination of ART clients’ hospital records and discussions with ART Programmes In-charges suggested that, indeed, more women than men are currently on ART in Ghana. We believe this situation could potentially be explained by a number of factors. Generally, the biological make-up of women makes them more susceptible to HIV infection than men. HIV is more readily transmitted from men to women than women to men, with explanations related to the larger surface area of the vagina and frequency of tears during sexual intercourse [24]. While this biological vulnerability is indisputable, it has been recognised that stressful factors such as social and economic vulnerability of women in Africa often drive and sustain patterns of unsafe sexual practices that place women at risk of HIV [25]. This may partly explain why more women are currently infected with HIV than men both in Ghana and across the Sub-Saharan Africa region [25, 26]. This may in turn explain why more women than men are on ART. Also, improved screening of women for HIV during pregnancy in Ghana has meant that many more HIV+ women are detected and enrolled on ART than men. Coupled with current levels of low counseling and testing among men [27], it is plausible to think that more women would dominate the list of ART clients. In addition, HIV+ men have been found elsewhere to be less willing to enroll on ART programmes partly because of masculinity and gender role/identity conflicts and partly because of shame and stigma [28, 29]. It is therefore possible that many HIV+ men in Ghana may be abstaining from ART programmes for similar reasons. In this regard, there may be the need for further research to understand why more women than men are receiving HIV treatment at ART clinics in Ghana. In the meantime, our findings here would suggest the need for interventions to also screen men as well as reach out to HIV+ men with available treatments.

All (100%) the HIV-positive persons interviewed in this study were linked to ART. Consistent with previous research elsewhere [29], we found that the reasons why participants were on ART ranged from beliefs that ART will suppress the HIV virus, desire to maintain good health and prolong life, and desire not to infect unborn children, to desire both to avoid death, and to become good therapeutic citizens (abide by doctors’ advice). That all participants recognised the benefits of ART and wanted to be on ART clearly presents a golden opportunity for the government of Ghana, the Ghana Health Service, healthcare providers and international partners to build on this enthusiasm and initiate and/ or scale-up programmatic and treatment interventions to reach all HIV+ persons who need treatment. This could include the provision of better and expanded screening services especially to rural and hard-to-rich populations, the creation of more HIV treatment centres, and the resourcing of such centres with trained professional caregivers and essential commodities. Of course, given the limited financial and human resources that are usually dedicated to healthcare provisioning in Ghana, implementing these recommendations can put additional pressure on the healthcare system. However, the benefits of this additional investment could be enormous as modest but effective ART coverage has been shown to substantially reduce morbidity and mortality related to HIV/AIDS infection as well as HIV transmission risks [1–5].

Despite the fact that all the HIV-positive persons sampled in this study were already linked to ART, 63.3% of them reported experiencing at least one major barrier in accessing ART. While the 36.7% who reported experiencing no challenge in accessing HIV treatment services is an encouraging sign that Ghana is probably doing some things right, the fact that majority of the ART clients are experiencing challenges in accessing care must be cause for concern. Among the first most important barriers the participants in this study reported were delays associated with receiving care from treatment centres (10.9%), long distance to treatment centres (1.1%), high financial costs associated with accessing and receiving ART (12.6%), job insecurity arising from regular leave of absence to receive ART (2%), shortage of drugs and other commodities (13.3%), fear of side effects of taking ARVs (4.8%), and stigma (1.3%). These barriers are very similar to what has been documented in other previous studies elsewhere [7, 14, 17–21] and in Ghana more specifically [9, 11].

A substantial number of participants (10.9%) reported delays associated with receiving care from treatment centres as a major barrier. Delays came from long waiting times before care was received. This long waiting time is often the result of limited numbers of trained healthcare personnel and ART centres. Compounding the problem is the fact that only specific days in a month are dedicated to all ART clients to receive care. The lack of individualised booking system to receive care has meant that large numbers of patients are brought to treatment centres to receive care at the same time. Very often, the limited healthcare staff at most of the treatment centres is not able to promptly provide care to all clients, leading to delays. As other researchers have found, most clinics are under-resourced with long waiting queues – all of which challenge the success in retaining HIV patients in chronic care [5, 29, 30]. While these delays are part of the realities of the healthcare delivery systems in most resource limited settings and may therefore not be unique to ART clinics, they are a major disincentive for PLH to access ART. As a number of our research participants reported, these delays are very concerning for ART clients who are employed in the formal sector, and who often require permission to absent themselves from work in order to attend ART clinic sessions. Therefore, spending long hours at treatment centres instead of work places could offer employers reasons to replace such employees. Our findings here would therefore suggest the need for interventions to address these delays and other related barriers (see Table 3).

**Table 3:** Potential ways to address barriers encountered in accessing ART

Barrier	Possible way(s) to address barrier
Delay before care is received	- Establish more ART centres - Train more caregivers - Institute an individualised booking system so ART clients can choose different days to receive care
Long distance to treatment centres	- Establish more ART centres - Implement occasional outreach programmes to care for ART clients - Institute travel costs reimbursement schemes - Create family, community, and peer support groups
Financial challenges	- Establish targeted social and economic safety net interventions for HIV+ persons e.g. conditional cash transfer programmes - Include HIV+ persons, especially ART clients in LEAP programme - Create family, community, and peer support groups - Institute free/subsidised nutrition supplementation interventions - Institute travel costs reimbursement schemes
Shortage of drugs and other commodities	- Allocate more financial resources, especially financial resources to ART - Prevent wastage in the use of drugs and other commodities - Reduce inefficiencies in management and use of medicines
Fear of side effects	- Educate ART client on how to properly administer medications - Educate ART clients on possible side effects of different treatment regimens and how to minimise potential side effects
Stigmatisation	- Educate public on HIV/AIDS using both print and electronic media, including social media - Institute anti-stigma interventions using community based and led strategies

In addition to increasing the number of caregivers at ART clinics, it might be helpful to design and institute an individualised booking system whereby individual clients could choose different days during the month to attend ART clinics. This could reduce the numbers of ART clients seeking care at any particular point in time to manageable sizes. This may reduce the overcrowding and long waiting time that are currently associated with ART clinics. The recommendations here are supported by previous evidence from elsewhere that suggest that increasing ART centres and health workforce can reduce delays that are associated with receiving ART [12].

Several of the participants (12.6%) also reported cost as a barrier to accessing care. While the current direct cost of ART in Ghana is relatively negligible, many HIV+ persons are still not able to afford to pay this cost and other additional indirect costs. This is partly because current approaches to providing care include multiple clinic visits, which are costly for the patient in terms of money and time. Consequently, patients who are poor and lack resources for transport might opt to not attend clinic. This could adversely affect adherence to treatment and may cause relapse. In addition to the inclusion of PLH in social and economic safety net programmes as we have suggested above, the institutionalisation of a travel costs reimbursement scheme might be useful. Elsewhere, similar schemes have been reported to enhance access to ART as well as increase ART adherence rates among HIV+ persons [13]. In the context of Ghana, such a scheme could be used to reimburse travel costs for ART clients, especially those from rural areas who often travel long distances to access ART. Also, there is the need to generate social support for ART clients at the levels of peers, family and community. Such an intervention has been shown to greater improve access to ART and as well enhance adherence to ART [31]. In the context of Ghana, such social support interventions could be modelled along the lines of the directly observed treatment (DOTs) methods which have been successfully used in the treatment and management of tuberculosis (TB). As DOTs has successfully been used in Ghana in the context of TB, we believe a similar approach in the area of ART has the potential to succeed.

Shortage of drugs and other commodities was also reported as a major barrier to effective ART access. This indeed is one of the major challenges confronting effective HIV/AIDS treatment across low- and middle-income countries, and a number of researchers and policy documents have recently acknowledged it [4–7]. In the presence of shortage of ARVs and other essential consumables, the full benefits of ART may not be realised. This would suggest the need for more resources to be allocated to purchasing and stocking ART clinics with sufficient quantities of essential medicines and commodities. This should also be accompanied by strategies to prevent waste and inefficiencies in the management and use of medicines and essential commodities through such practices as over prescription and corruption (e.g. stealing or embezzlement of medicines from public ART clinics for personal profit). Indeed, there is evidence to suggest that countries that have made significant progress in rolling out ART and reducing HIV infection have done so with significant investment of both human and financial resources [12, 13, 32]. Such countries have also effectively prevented wastage, theft of medicines and corruption in the management of ART programmes [12, 13, 32].

Additional barriers that participants reported included fear of side effects of taking ARVs and stigma. We believe patient education on how to appropriately administer medications as well as possible side effects of different treatment regimen may help reduce both the side effects of drugs and the fear that some patients harbour. Similarly, continues public education and interventions to de-stigmatise HIV/AIDS and reduce stigma and discrimination against PLH may help increase uptake of ART. As previous studies have shown, both patient and public education can greatly improve adherence to ART by reducing anxieties about of side effects of ARVs as well as minimise stigma against HIV+ persons [13, 20, 32].

Taken together, the findings presented in this paper highlight an urgent need for interventions to address different individual level and structural barriers to ART access so as to optimise the full benefits of ART. The findings and the accompanying recommendations should however be read against the backdrop of certain limitations. The research reported in this paper was conducted at four ART clinics in southern Ghana. Therefore, the limitation of applying the findings in other parts of the country is acknowledged. This is more so because HIV+ persons are not a homogenous group, hence the perspectives of those interviewed in this study might be different from what pertains elsewhere in Ghana. In this regard, large-scale quantitative and qualitative studies might be required to provide a more holistic picture about the challenges HIV+ persons face in accessing ART services at the national or regional levels. Finally, there are a number of characteristics not captured in the baseline demographics but have been shown to be associated with access/ retention on ART. These include active illicit drug/ alcohol use, mental illness, homelessness, viral load, type of ART regimen prescribed and dosing, criminal justice history, domestic and/ or interpersonal violence. These limitations notwithstanding, important lessons can be drawn from the findings in this paper to inform policies and interventions that seek to promote greater access to HIV/AIDS treatment for HIV+ persons. In particular, the results of the study provide useful pointers for policymakers and healthcare providers within Ghana and across the world to participate in designing innovative interventions to redress the remaining barriers that PLH face in accessing needed treatment.

## Conclusions

Results from this study indicate that a number of structural and individual level barriers still act, alone or in concert, to hinder HIV+ persons’ access to ART in Ghana, even in situations where these services are available. Efforts to provide and scale-up ART to all HIV+ persons and ensure adherence must also be accompanied by interventions that address these remaining structural and individual level access barriers. Among other interventions (see Table 3), we recommend the institutionalisation of individual booking/appointment system of care, and a travel reimbursement scheme for ART clients, more investment to train more staff, purchase and stock ART clinics with essential drugs and commodities as well as patient and public education, to help address these challenges.

## Additional file

Additional file 1: The Survey Questionnaire is included as an additional file. (DOCX 123 kb)

## Abbreviations

AIDS: Acquired Immune deficiency Syndrome
ART: Antiretroviral therapy
ARVs: Antiretroviral
CSPro: Census and Survey Processing System
DOT: Directly observed treatment
GHS: Ghana cedis
HIV: Human immunodeficiency virus
LEAP: Livelihoods empowerment against poverty
PLHIV: Persons living with Human Immunodeficiency Virus
RAs: Research assistants
SPSS: Statistical package for the social science
STI: Sexually transmitted infection
TB: Tuberculosis
WHO: World Health Organization
