# Supplementary material for: ART access-related barriers faced by HIV-positive persons linked to care in southern Ghana: a mixed method study
Source: BMC Infect Dis. 2016 Dec 7;16:738. doi: 10.1186/s12879-016-2075-0 (PMC5142337; doi:10.1186/s12879-016-2075-0)
Supplement: Additional file 1: — The Survey Questionnaire is included as an additional file. (DOCX 123 kb) [file 12879_2016_2075_MOESM1_ESM.docx]

**NON-PRESCRIPTION DRUGS USE AMONG GHANAIAN PERSONS PLHIVs**

**NOTE: Introduce study and administer consent here:**

| **Basic Information/tracking details** |
| --- |
| **101 Date of Interview: _____________ /_____________ / _______________**  **(101a) Day (101b) Month (101c) Year** |
| **102 Participant’s number:** |
| **103 Study site:(1) Atua (2) St Martin’s (3) Tema General Hospital (4) Fevers Unit, Korle Bu** |
| **104 Region: (1) Greater Accra (2) Eastern Region** |
|  |
| **105 Name of Research Assistant:** |
| **106 Phone number:** |
|  |
| **107 Start time: _____________ _____________ _______________**  **(109a) Hours (109b) Minutes (109c) AM/PM** |

**
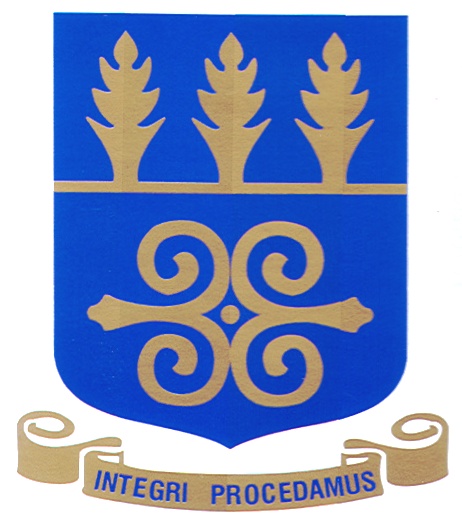

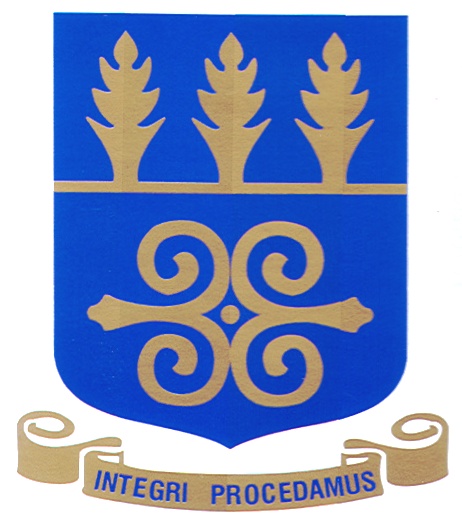

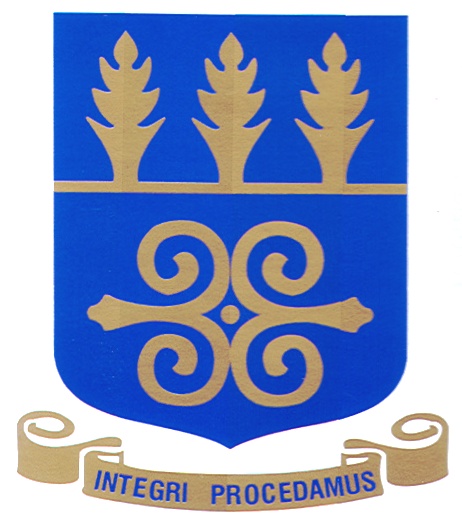

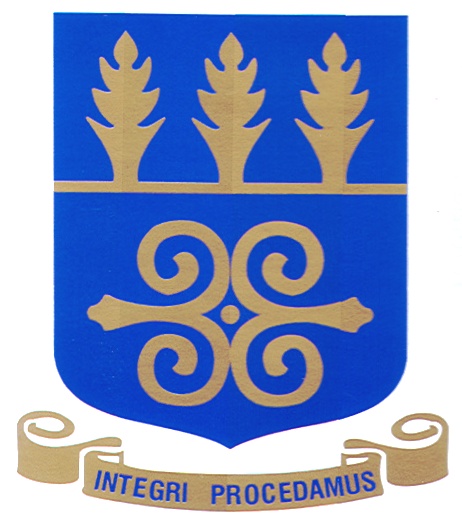
**

**SECTION A. BACKGROUND INFORMATION OF STUDY PARTICIPANT**

**A1 Age of respondent (In Years)………………………………………………**

**A2 Sex of respondent (M/F)……………………………………………….**

**A3. Marital status of respondent**

(1). Single (2). Married (3). Divorced/Separated (4). Widowed (5) Cohabiting

**A4. Religious affiliation of respondent:**

(1). None (2). Christian (3). Muslim (4) Traditionalist (5) Other……………..

**A5. Place of residence (may not be the same as where care is accessed):**

(1) Urban (2) Rural

**A6. Respondent’s level of education:**

(1). No formal education (1). Primary (2).JHS (3).SHS/Vocational/ (4) Post secondary education/Tertiary

**A7. Current Occupation of respondent:**

(1). Unemployed (2).Farmer (3). Teacher (4).Trader (5).Secretary/Accounts clerk

((6). Work in a hotel (7) Music industry (8) Marketing agent (9) Other………………………..

**A8. Does respondent have an Adherence monitor (1) YES (2) NO –(SKIP TO A. 10)**

**A9. Relationship to adherence monitor**

(1). Spouse (2).Parent (3).Nurse/other health worker (4).Other family member (5).Friend (6).Other……………….…….

**A10a. Reproductive health history: (Ask females only)**

1. Gravidity------------------------ (2) Parity--------------------------- (3) No. of children alive-------

A10b. If RESPONDENT IS A FEMALE INDICATE WHETHER SHE IS

( 1) Pregnant-----. (2) Lactating----- (3) Not pregnant ----

A11. What is the sex of your current child? [ASK THIS ONLY, IF SUBJECT HAS A CHILD UNDER 2 YEARS]

- 1. Male
  2. Female

A12. What is his/her age.................................. (months) –IF A14 IS APPLICABLE

A13. What is the size of your family? ………………………

###### B. HEALTH-SEEKING BEHAVIOR

**B1. Which health facilities are available in this area? (Multiple Response)**

(1) Clinics (2) Pharmacies/drug stores (3) indigenous healers (4) healing churches (5) other (specify) ……………………………….. (Probe)

**B2. Which of these have you accessed in the last 3 months?(Multiple Response)**

(a)Clinics (b) Pharmacies/drug stores (c) indigenous healers (d) healing churches (5) other specify …………………………………. (Probe)

**B3. What health problems did you take there**?.............................................................................................................................

………………………………………………………………………………………………………………………………

**B4. What kind of medications were you given??** ………………………………………………………………………………………………………………………………………………………………………………………………………………………………………………………………………………**B5. Why do you use these other facilities: [IF APPLICABLE]** ………………………………………………………………………………………………………………………………………..………………………………………………………………………………………………………………………………………**B7. When did you start receiving ART?**........................................................................... **[MONTH AND YEAR] (Get this from the patient folder)**

**B8. Identify problems they encounter receiving ART.**

1. ………………………………………………………………………………………………………………………………

b…………………………………………………………………………………………………………………………………

c…………………………………………………………………………………………………………………………

**Section C: MEDICATIONS OR SUBSTANCES BEING USED IN ADDITION TO ART.**

All medications (REVIEW HOSPITAL RECORDS, PATIENTS’ FOLDERS AND ALSO THROUGH INTERVIEWER). **(GET FROM PATIENT FOLDER OR APPOINTMENT CARD)**

(C1) ART **(list here all ART the patient takes)** **(Get this from the patient folder)**

--------------------------------------------------------------------------------------------------------------------------------------------

--------------------------------------------------------------------------------------------------------------------------------------------

----------------------------------------------------------------------------------------------------------------------------------------------

----------------------------------------------------------------------------------------------------------------------------------------------

-----------------------------------------------------------------------------------------------------------------------------------------------

(C2) Do you take other medications (both approved and non-approved in addition to the ART?

[ ] YES [ ]NO

(C2) Other approved/prescribed medications (GET FROM PATIENT FOLDER OR APPOINTMENT CARD)

-----------------------------------------------------------------------------------------------------------------------------------------------------

-----------------------------------------------------------------------------------------------------------------------------------------------------

-----------------------------------------------------------------------------------------------------------------------------------------------------

-----------------------------------------------------------------------------------------------------------------------------------------------------

-----------------------------------------------------------------------------------------------------------------------------------------------------

(C3) Do you take Traditional/herbal medications ?

[ ] YES [ ]NO

If yes, list traditional/herbal medications (GET THESE THROUGH INTERVIEW)

-----------------------------------------------------------------------------------------------------------------------------------------------------

-----------------------------------------------------------------------------------------------------------------------------------------------------

-----------------------------------------------------------------------------------------------------------------------------------------------------

-----------------------------------------------------------------------------------------------------------------------------------------------------

-----------------------------------------------------------------------------------------------------------------------------------------------------

(C4) Other drugs (LIST HERE OTHER DRUGS BEING TAKEN)

-----------------------------------------------------------------------------------------------------------------------------------------------------

-----------------------------------------------------------------------------------------------------------------------------------------------------

-----------------------------------------------------------------------------------------------------------------------------------------------------

-----------------------------------------------------------------------------------------------------------------------------------------------------

(C5) Do you take Nutrient Supplments? [ ] YES [ ]NO

If yes list here all Nutrient supplements being taken (CHECK FROM FOLDER AND ALSO THROUGH INTERVIEW)

-----------------------------------------------------------------------------------------------------------------------------------------------------

-----------------------------------------------------------------------------------------------------------------------------------------------------

-----------------------------------------------------------------------------------------------------------------------------------------------------

**Section D: Motivators for use of medications/substances (Refer to section C)**

*Qualitatively assess why the medications/substance mentioned above are being used:*

(D1) Ask patient why he or she takes the various medications/drugs/substances listed in SECTION C – C1 to C5. Probe for reasons for each of them.

-----------------------------------------------------------------------------------------------------------------------------------------------------(D1A) REASONS WHY THEY TAKE ART

--------------------------------------------------------------------------------------------------------------------------------------------

--------------------------------------------------------------------------------------------------------------------------------------------

----------------------------------------------------------------------------------------------------------------------------------------------

----------------------------------------------------------------------------------------------------------------------------------------------

-----------------------------------------------------------------------------------------------------------------------------------------------

(D1B) REASONS WHY THEY TAKE other approved/prescribed medications

-----------------------------------------------------------------------------------------------------------------------------------------------------

-----------------------------------------------------------------------------------------------------------------------------------------------------

-----------------------------------------------------------------------------------------------------------------------------------------------------

-----------------------------------------------------------------------------------------------------------------------------------------------------

-----------------------------------------------------------------------------------------------------------------------------------------------------

(D1C) REASONS WHY THE TAKE Traditional/herbal medications

-----------------------------------------------------------------------------------------------------------------------------------------------------

-----------------------------------------------------------------------------------------------------------------------------------------------------

-----------------------------------------------------------------------------------------------------------------------------------------------------

-----------------------------------------------------------------------------------------------------------------------------------------------------

-----------------------------------------------------------------------------------------------------------------------------------------------------

(D1D) REASONS WHY THE TAKE DRUGS OTHER THAN THOSE LISTED ABOVE....

-----------------------------------------------------------------------------------------------------------------------------------------------------

-----------------------------------------------------------------------------------------------------------------------------------------------------

-----------------------------------------------------------------------------------------------------------------------------------------------------

(D1E) REASONS WHY THE TAKE NUTRIENT SUPPLEMENTS

-----------------------------------------------------------------------------------------------------------------------------------------------------

-----------------------------------------------------------------------------------------------------------------------------------------------------

-----------------------------------------------------------------------------------------------------------------------------------------------------

(D2) Qualitatively explore whether they are taking other medication and substance as a result of periodic shortages of ARVs,

-----------------------------------------------------------------------------------------------------------------------------------------------------

-----------------------------------------------------------------------------------------------------------------------------------------------------

-----------------------------------------------------------------------------------------------------------------------------------------------------

-----------------------------------------------------------------------------------------------------------------------------------------------------

(D3) Explore whether it is due to cost of ARVs

-----------------------------------------------------------------------------------------------------------------------------------------------------

-----------------------------------------------------------------------------------------------------------------------------------------------------

-----------------------------------------------------------------------------------------------------------------------------------------------------

-----------------------------------------------------------------------------------------------------------------------------------------------------

(D4) Explore whether it is due to side effects associated with ARVs

-----------------------------------------------------------------------------------------------------------------------------------------------------

-----------------------------------------------------------------------------------------------------------------------------------------------------

-----------------------------------------------------------------------------------------------------------------------------------------------------

-----------------------------------------------------------------------------------------------------------------------------------------------------

(D5) Explore whether it is due to the perceived efficacy of alternative medications

-----------------------------------------------------------------------------------------------------------------------------------------------------

-----------------------------------------------------------------------------------------------------------------------------------------------------

-----------------------------------------------------------------------------------------------------------------------------------------------------

(D6) ASK patient whether they will still come for ART if they were to be PENALIZED for taking medications that their Doctors do not approve. YES [ ] NO [ ]

GIVE REASONS FOR EITHR YES, OR NO

---------------------------------------------------------------------------------------------------------------------------------------------------------------------------------------------------------------------------------------------------------------------------------------------------------------------------------------------------------------------------------------------------------------------------------------------------------------------------------

D7. Do you adhere to your ARV regimen? [ ] YES [ ]NO

D8. Do you take the correct number of pills always? [ ] YES [ ]NO

D9. Do you take your medications at the right time? [ ] YES [ ]NO

D 10. Do you take your medicines with food? [ ] YES [ ]NO

D 11. If no, to any of the above, please ask respondent to give reasons why

………………………………………………………………………………………………………………………………………………………………………………………………………………………………………………………………………………………………………………………………………………………………………………………………………………………

D12. In past month, how many times did you miss taking your ARV medications? …………………………………………. (WRITE THE TOTAL NUMBER OF TIMES, THAT PATIENT MISSED HIS/HER MEDICATIONS LAST MONTH)

**Section E: MODE/MANNER OF ADMINISTRATION MEDICATIONS/SUBSTANCES:**

*Assessing how the medications are administered*:

(E1) FIND OUT FROM PATIENT whether THE OTHER medications are administered together with ART at the same time, and why

YES [ ] NO [ ]

GET REASONS FOR EITHER OF THE ABOVE RESPONSES HERE

-----------------------------------------------------------------------------------------------------------------------------------------------------------------------------------------------------------------------------------------------------------------------------------------------------------------------------------------------------------------------------------------------------------------------------------------------------------------------------------------------------------------------------------------------------------------------------------------------------------------------------------------------------------------------------------------------------------------------------------------------------

(E2) FIND OUT FROM PATIENT whether medications are substituted for ARVs (taken in place of ARVs)

YES [ ] NO [ ]

GET REASONS FOR EITHER OF THE ABOVE RESPONSES HERE

-----------------------------------------------------------------------------------------------------------------------------------------------------------------------------------------------------------------------------------------------------------------------------------------------------------------------------------------------------------------------------------------------------------------------------------------------------------------------------------------------------------------------------------------------------------------------------------------------------------------------------------------------------------------------------------------------------------------------------------------------------

(E3) FIND OUT FROM PATIENT whether medications are administered independent or with other substances (list additional substances or media)

YES [ ] NO [ ]

GET REASONS FOR EITHER OF THE ABOVE RESPONSES HERE

------------------------------------------------------------------------------------------------------------------------------------------------------------------------------------------------------------------------------------------------------------------------------------------------------------------------------------------------------------------------------------------------------------------------------------LIST THOSE ADDITIONAL SUBSTANCES HERE

----------------------------------------------------------------------------------------------------------------------------------------------------------------------------------------------------------------------------------------------------------------------------------------

----------------------------------------------------------------------------------------------------------------------------------------------------------------------------------------------------------------------------------------------------------------------------------------

(E4) FIND OUT FROM PATIENT THE nature and formulations medications/substances used (WHETHER Powder, liquid, solution, TABS, PILLS, etc)

---------------------------------------------------------------------------------------------------------------------------------------------------------------------------------------------------------------------------------------------------------------------------------------------------------------------------------------------------------------------------------------------------------------------------------------------------

(E5). FIND OUT FROM PATIENT other challenges respondents face accessing and adhering to ART

----------------------------------------------------------------------------------------------------------------------------------------------------------------------------------------------------------------------------------------------------------------------------------------------------------------------------------------------------------------------------------------------------------------------------------------------------------------------------------------------------------------------------------------------------------------------------------------------------------------------------------------------------------------------------------------------------------------------------

--------------------------------------------------------------------------------------------------------------------------------------------------------------------------------------------------------------------------------------------------------------------------------------------------------------------------------------------------------------------------------------------------------------------------------------------------------------------------------------------------------------------------------------------------------------------------------

(E6) Ask respondent to suggest ways to improve ARV treatment at this facility

-----------------------------------------------------------------------------------------------------------------------------------------------------------------------------------------------------------------------------------------------------------------------------------------------------------------------------------------------------------------------------------------------------------------------------------------------------------------------------------------------------------------------------------------------------------------------------------------------------------------------------------------------------------------------------------------------------------------------------------------------------------------------------------------------------------------------------------------------------------------------------------------------------------------------------------------------------------------------------------------------------------------------------------------------------------------------------------------------------------------------------------------------------------------------------------------------------------------------------------------------------------------------------------------------------------------------------------------------------------------------------------------------------------------------------------------------------------------------------------------------------------------------------------------------------------------------------------------------------------------------------------------------------------

**SECTION F:RAPID DIETARY SURVEY**

**F1. Do you CURRENTLY have problems with eating in general?**

1). NO (2). YES

**F2. IF YES, What ARE THESE problems?**

--------------------------------------------------------------------------------------------------------------------------------------------

--------------------------------------------------------------------------------------------------------------------------------------------

--------------------------------------------------------------------------------------------------------------------------------------------

**F3. What ARE YOU CURRENTLY DOING to address these problems?**

------------------------------------------------------------------------------------------------------------------------------------------------------------------------------------------------------------------------------------------------------------------------------------------------------------------------------------------------------------------------------------------------------------------------------------------------------------------------------------------------------------------------------------------------------------------------------------------------------------------------------------------------------------------------------------------------------------------------

**F4**. **Do you use nutrient supplements?**1). NO (2). YES

**F5. If yes let respondent specify the various supplements being used**

----------------------------------------------------------------------------------------------------------------------------------------------------------------------------------------------------------------------------------------------------------------------------------------------------------------------------------------------------------------------------------------------------------------------------------------------------------------------------------------------------------------------------------------------------------------------------

**F6. Have you stopped consuming any food item lately or after your DIAGNOSIS?**

(1). NO (2). YES

**F7. If YES, Name these items and the reasons why you stopped consuming them**

| **ITEM** | **REASONS** |
| --- | --- |
|  |  |
|  |  |
|  |  |
|  |  |

**F8. Have you started consuming any food item that you were not consuming before YOUR DIAGNOSIS?**

(1). NO (2). YES

**F9. Name these items and the reasons why you have started consuming them.**

| ITEM | REASONS |
| --- | --- |
|  |  |
|  |  |
|  |  |
|  |  |

**F10. How many adults (18+ years) and children (6-17 years) in your household did not consume any food yesterday**?

Number of adults who did not consume any food yesterday………………………..

Number of children who did not consume any food yesterday………………………..

What is he total number of adults and children in your household………………………..

**F11. Coping strategy assessment**

In the past three months, how frequently did you or your household resort to using one or more of the following strategies in order to have access to food? **CIRCLE ONLY ONE ANSWER PER STRATEGY.**

|  |  | Never | Seldom  (1-3 days/month) | Sometimes  (1-2 days /week) | Often  (3-6 days a week) | Daily |
| --- | --- | --- | --- | --- | --- | --- |
|  |  | 1 | 2 | 3 | 4 | 5 |
| **1** | Skip entire days without eating? | 1 | 2 | 3 | 4 | 5 |
| **2** | Limit portion size at mealtimes? | 1 | 2 | 3 | 4 | 5 |
| **3** | Reduce number of meals eaten per day? | 1 | 2 | 3 | 4 | 5 |
| **4** | Borrow food or rely on help from friends or relatives? | 1 | 2 | 3 | 4 | 5 |
| **5** | Rely on less expensive or less preferred foods? | 1 | 2 | 3 | 4 | 5 |
| **6** | Purchase/borrow food on credit? | 1 | 2 | 3 | 4 | 5 |
| **7** | Gather unusual types or amounts of wild food / hunt? | 1 | 2 | 3 | 4 | 5 |
| **8** | Harvest immature crops (e.g. green maize)? | 1 | 2 | 3 | 4 | 5 |
| **9** | Send household members to eat elsewhere? | 1 | 2 | 3 | 4 | 5 |
| **10** | Send household members to beg? | 1 | 2 | 3 | 4 | 5 |
| **11** | Reduce adult consumption so children can eat? | 1 | 2 | 3 | 4 | 5 |
| **12** | Rely on casual labour for food? | 1 | 2 | 3 | 4 | 5 |

**Section G: ANTHROPOMETRIC MEASUREMENTS**

***G1. Current weight in kg****……………………………………………………………*

***G2. Height in cm:*** *………………………………………………………………….......*

***G3. MUAC/cm****……………………………………………………………………..........*

**SECTION H: CLINICAL AND LABORATORY INFORMATION**

**H1. DATE HIV first diagnosed [MONTH…………….. AND YEAR………………]**

**H2 HIV disease state**

(1) HIV Positive with no AIDS (2) HIV Positive with AIDS

**H3 Hemoglobin in g/dl:** ………………………………………………………………………….

**H4 CD4+ cell count (cells/mm^3^)** …………………………………………………………..

**THANK PARTICIPANT, & END INTERVIEW IF PARTICIPANT IS NOT A FEMALE**
